# Feeding oxidized fat during pregnancy up-regulates expression of PPARα-responsive genes in the liver of rat fetuses

**DOI:** 10.1186/1476-511X-6-6

**Published:** 2007-03-12

**Authors:** Robert Ringseis, Anke Gutgesell, Corinna Dathe, Corinna Brandsch, Klaus Eder

**Affiliations:** 1 Institut für Agrar- und Ernährungswissenschaften, Martin-Luther-Universität Halle-Wittenberg, Emil-Abderhalden-Straße 26, D-06108 Halle/Saale, Germany

## Abstract

**Background:**

Feeding oxidized fats causes activation of peroxisome proliferator-activated receptor α (PPARα) in the liver of rats. However, whether feeding oxidized fat during pregnancy also results in activation of PPARα in fetal liver is unknown. Thus, this study aimed to explore whether feeding oxidized fat during pregnancy causes a PPARα response in fetal liver. Two experiments with pregnant rats which were administered three different diets (control; oxidized fat; clofibrate as positive control) in a controlled feeding regimen during either late pregnancy (first experiment) or whole pregnancy (second experiment) were performed.

**Results:**

In both experiments pregnant rats treated with oxidized fat or clofibrate had higher relative mRNA concentrations of the PPARα-responsive genes acyl-CoA oxidase (ACO), cytochrome P_450 _4A1 (CYP4A1), L-type carnitin-palmitoyl transferase I (L-CPT I), medium-chain acyl-CoA dehydrogenase (MCAD), and long-chain acyl-CoA dehydrogenase (LCAD) in the liver than control rats (*P *< 0.05). In addition, in both experiments fetuses of the oxidized fat group and the clofibrate group also had markedly higher relative mRNA concentrations of ACO, CYP4A1, CPT I, MCAD, and LCAD in the liver than those of the control group (*P *< 0.05), whereas the relative mRNA concentrations of PPARα, SREBP-1c, and FAS did not differ between treatment groups. In the second experiment treatment with oxidized fat also reduced triacylglycerol concentrations in the livers of pregnant rats and fetuses (*P *< 0.05).

**Conclusion:**

The present study demonstrates for the first time that components of oxidized fat with PPARα activating potential are able to induce a PPARα response in the liver of fetuses. Moreover, the present study shows that feeding oxidized fat during whole pregnancy, but not during late pregnancy, lowers triacylglycerol concentrations in fetal livers.

## Background

In recent years the contribution of dietary oxidized fats to total energy intake has markedly increased in industrialized countries, mainly, due to the increasing consumption of fast food which contain significant amounts of these heated and processed fats. We and others have shown that oxidized fats are strong activators of peroxisome proliferator-activated receptor α (PPARα) [1-3], a ligand-activated transcription factor that belongs to the family of nuclear receptors. PPARα is centrally involved in the regulation of lipid homeostasis in the liver and is essential for normal liver function [4]. Upon activation of PPARα by a ligand, which includes the fibrate class of hypolipidemic drugs such as clofibrate or fenofibrate, fatty acids, and eicosanoids, the transcription of genes containing a PPAR response element (PPRE) in its promoter region is induced. Typical PPARα-responsive genes in the liver, which are also up-regulated by oxidized fats and its components such as oxidized fatty acids (e.g. hydroperoxy-fatty acids, cyclic fatty acids) [1-3,5,6], include a wide array of genes that are involved in peroxisomal and mitochondrial fatty acid β-oxidation such as L-type carnitin-palmitoyl transferase I (L-CPT I), acyl-CoA oxidase (ACO), cytochrome P_450 _4A1 (CYP4A1), medium-chain acyl-CoA dehydrogenase (MCAD), and long-chain acyl-CoA dehydrogenase (LCAD) [7,8]. PPARα activation by oxidized fat was also shown to cause liver enlargement due to peroxisome proliferation and reduce triacylglycerol concentrations in liver, plasma, and very low-density lipoproteins in rats [3,9,10]. In part, the triacylglycerol-lowering effect of oxidized fat in the rat is presumably also due to the observed reduction in mRNA concentrations and activities of lipogenic enzymes such as fatty acid synthase (FAS) [9].

During pregnancy treatment of rats with the pharmacological PPARα activator clofibrate has been shown to cause proliferation of peroxisomes [11,12], induction of peroxisomal enzyme activities [13], and induction of CYP4A1 in fetal liver [14] indicating that clofibrate is capable of activating PPARα transplacentally. In addition, pathological changes in newborn rats born to mothers treated with clofibrate during pregnancy [15], and an impaired fetal growth of fenofibrate-treated pregnant rats have been reported [16]. Moreover, in rat and mouse liver epithelial cells treatment with the PPARα agonist WY-14,643 caused up-regulation of proto-oncogenes [17-19], which has been attributed to the hepatocarcinogenic effect of peroxisome proliferators in rodents [18]. However, whether feeding oxidized fat to pregnant rats also results in activation of PPARα and up-regulation of PPARα target genes in fetal livers is unknown from the literature. Moreover, it is unknown whether components of oxidized fats such as oxidized fatty acids are able to substantially pass the placenta and enter the fetus, because it has been shown that the transplacental transport of fatty acids from the maternal diet is highly selective for individual fatty acids, e.g. long-chain polyunsaturated fatty acids such as docosahexaenoic acid and arachidonic acid are preferentially transported through the placenta at the expense of other less important fatty acids [20-22].

Therefore, the present study aimed to explore whether feeding oxidized fat during pregnancy causes a PPARα response in fetal liver as estimated by the up-regulation of typical PPARα-responsive genes such as ACO, L-CPT I, CYP4A1, MCAD, and LCAD and whether the induction of fatty acid catabolism might also affect fetal hepatic triacylglycerol concentrations. We also analyzed the mRNA abundance of the lipogenic transcription factor sterol regulatory-element binding protein (SREBP)-1c and its target gene FAS, because in previous studies administration of oxidized fats has also been demonstrated to reduce mRNA expression of lipogenic enzymes [9]. To address possible adverse effects of treatment with oxidized fat, we also determined the mRNA abundance of the proto-oncogenes c-myc, c-jun, and c-fos.

Since the duration of administration of oxidized fat during pregnancy might also influence the effect on lipid metabolism in the fetal liver, we performed two experiments which varied in the duration of administration of the oxidized fat. In the first experiment we investigated the effect of short-term administration (last 5 d of pregnancy) of oxidized fat on the PPARα response in maternal and fetal liver, whereas in the second experiment the effect of long-term administration (whole pregnancy) was studied.

## Results

### Fatty acid composition and concentrations of lipid peroxidation products in the experimental fats

The oxidized fat in the short-term experiment had lower proportions of polyunsaturated fatty acids (C18:2) but higher proportions of saturated (C16:0, C18:0) and monounsaturated (C18:1) fatty acids compared to the control fat due to the heat treatment of sunflower oil during preparation of the oxidized fat (Table 1). In the long-term experiment the proportions of C18:2 and C18:1 in the oxidized fat were similar to those in the control fat due to adjustment of fatty acid composition of the control fat, whereas the proportions of saturated fatty acids were lower in the oxidized fat compared to the control fat (Table 2). In both experiments the oxidized fat had higher concentrations of peroxides, TBARS, and conjugated dienes, and a higher acid value than the fat of the control group. The concentrations of lipid peroxidation products (peroxide value and conjugated dienes) in the control fat were higher in the long-term experiment than in the short-term experiment. This is probably due to the fact that fats in the long-term experiment were included into a semi-synthetic diet which contained pro-oxidant minerals such as iron or copper whereas fats in the short-term experiment were administered the rats directly by gastric tube.

**Table 1:** Characteristics of the experimental fats of the short-term experiment

Treatment group	Control	Clofibrate	Oxidized fat^1^
Fat	SFO^2^		oxidized SFO^2^
Major fatty acids, g/100 g total fatty acids			
C16:0	6.1		9.1
C18:0	3.4		5.3
C18:1 (n-9)	32.6		38.3
C18:2 (n-6)	56.6		44.7
Peroxidation products			
Peroxide value, mEq O_2_/kg	3.0		379
Acid value, g KOH/kg	0.4		5.8
Conjugated dienes, mmol/kg	< 0.1		274
TBARS^2^, mmol/kg	1.1		13.1

^1^Prepared by heating at a temperature of 60°C for 25 d.

^2^Abbreviations: SFO, sunflower oil; TBARS, thiobarbituric acid-reactive substances.

**Table 2:** Characteristics of the experimental fats of the long-term experiment

Treatment group	Control	Clofibrate	Oxidized fat
Fat	SFO^2 ^: lard (54:46)		oxidized SFO^2^
Major fatty acids, g/100 g total fatty acids			
C16:0	14.9		10.2
C18:0	9.5		6.2
C18:1 (n-9)	28.9		34.4
C18:2 (n-6)	42.0		44.2
Peroxidation products			
Peroxide value, mEq O_2_/kg	6.6		230
Conjugated dienes, mmol/kg	12.3		139
TBARS^2^, mmol/kg	1.0		19

^1^Prepared by heating at a temperature of 60°C for 25 d.

^2^Abbreviations: SFO, sunflower oil; TBARS, thiobarbituric acid-reactive substances.

### Final body weights and relative liver weights of pregnant rats

Body weight development was not affected by dietary treatment in both experiments due to the controlled feeding regimen applied. In the short-term experiment, final body weights of pregnant rats at d 21 of pregnancy did not differ between the three treatment groups (control, 300 ± 28 g; oxidized fat, 299 ± 24 g; clofibrate, 307 ± 17 g, mean ± SD). Pregnant rats treated with clofibrate or oxidized fat had higher relative liver weights than control rats (*P *< 0.05; control, 3.6 ± 0.2 g/100 g body weight; oxidized fat, 4.4 ± 0.3 g/100 g body weight; clofibrate, 4.9 ± 0.2 g/100 g body weight).

In the long-term experiment, final body weights of pregnant rats at d 21 of pregnancy were also not different between treatment groups (control, 342 ± 35 g; oxidized fat, 331 ± 40 g; clofibrate, 328 ± 18 g). Relative liver weights of pregnant rats treated with clofibrate or oxidized fat were also higher than those of control rats (*P *< 0.05; control, 3.4 ± 0.2 g/100 g body weight; oxidized fat, 4.4 ± 0.5 g/100 g body weight; clofibrate, 4.5 ± 0.3 g/100 g body weight).

### Relative mRNA concentrations of PPARα and PPARα-responsive genes in livers of pregnant rats and fetuses

**Figure 1:**
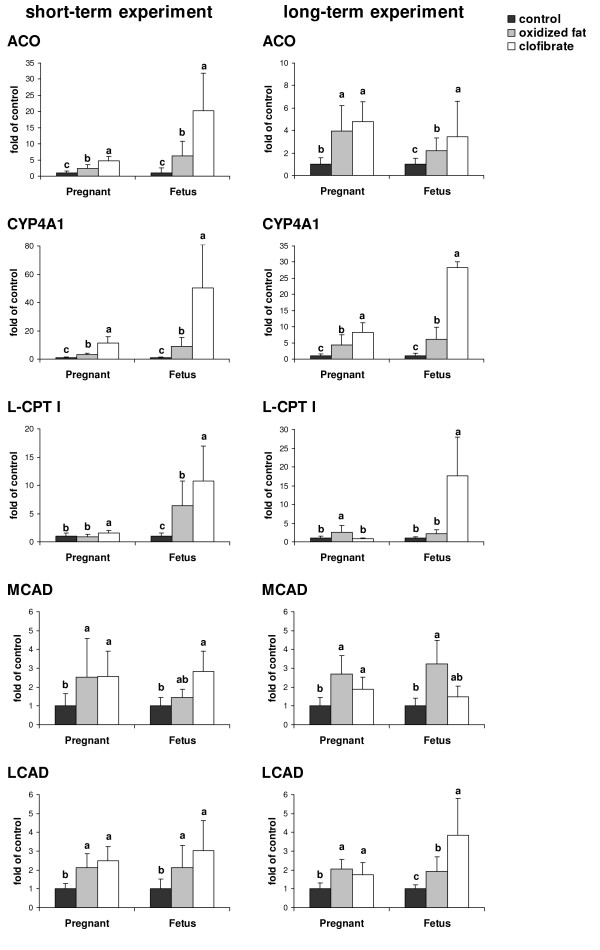
**Effects of treatment on relative mRNA concentrations of PPARα-responsive genes in livers of pregnant rats and fetuses**. Effect of short-term (d 16 – d 21 of pregnancy, left) and long-term (d 1 – d 21 of pregnancy, right) administration of three different diets (control, oxidized fat, clofibrate) during pregnancy on relative mRNA concentrations of ACO, CYP4A1, CPT I, MCAD, and LCAD in the liver of pregnant rats and fetuses at d 21 of pregnancy. Left, Bars represent mean ± SD (n = 9/group). Right, Bars represent mean ± SD (n = 12/group). Bars marked without a common superscript letter differ (*P *< 0.05). Results from one-way ANOVA (*P*-values): short-term experiment: ACO, *P *= 0.0001 (pregnant), *P *= 0.001 (fetus); CYP4A1, *P *= 0.0001 (pregnant), *P *= 0.002 (fetus); L-CPT I, *P *= 0.013 (pregnant), *P *= 0.012 (fetus); MCAD, *P *= 0.042 (pregnant), *P *= 0.001 (fetus); LCAD, *P *= 0.001 (pregnant), *P *= 0.03 (fetus); long-term experiment: ACO, *P *= 0.0001 (pregnant), *P *= 0.015 (fetus); CYP4A1, *P *= 0.0001 (pregnant), *P *= 0.001 (fetus); L-CPT I, *P *= 0.016 (pregnant), *P *= 0.011 (fetus); MCAD, *P *= 0.001 (pregnant), *P *= 0.019 (fetus); LCAD, *P *= 0.001 (pregnant), *P *= 0.001 (fetus).

In the short-term experiment, relative mRNA concentrations of PPARα in the liver of pregnant rats and fetuses were not different between treatment groups (pregnant rats: 1.00 ± 0.49, control group; 1.02 ± 0.48, oxidized fat group; 1.26 ± 0.22, clofibrate group; *P *= 0.439; fetuses: 1.00 ± 0.29, control group; 1.29 ± 0.50, oxidized fat group; 1.15 ± 0.74, clofibrate group; *P *= 0.653). Pregnant rats treated with oxidized fat had 2.4-, 3.0-, 2.5- and 2.1-fold higher relative mRNA concentrations of ACO, CYP4A1, MCAD, and LCAD, respectively, in the liver than control rats (*P *< 0.05; Fig. 1). The relative mRNA concentration of L-CPT I did not differ between both groups of rats. Treatment of pregnant rats with clofibrate resulted in 4.8-, 11-, 1.6-, 2.5- and 2.4-fold higher relative mRNA concentrations of ACO, CYP4A1, L-CPT I, MCAD, and LCAD, respectively, in the liver compared to control treatment (*P *< 0.05). Fetuses of the oxidized fat group had 6.3-, 9.0-, 6.4-, 1.5- and 2.1-fold higher relative mRNA concentrations of ACO, CYP4A1, L-CPT I, MCAD, and LCAD respectively, in the liver than those of the control group (*P *< 0.05), whereas fetuses of the clofibrate group had 20-, 51-, 12-, 2.8- and 3.0-fold higher relative mRNA concentration of ACO, CYP4A1, L-CPT I, MCAD, and LCAD, respectively in the liver than those of the control group (*P *< 0.05).

In the long-term experiment, relative mRNA concentrations of PPARα in the liver of pregnant rats and fetuses also did not differ between treatment groups (pregnant rats: 1.00 ± 0.49, control group; 1.56 ± 0.99, oxidized fat group; 0.63 ± 0.29, clofibrate group; *P *= 0.053; fetuses: 1.00 ± 0.70, control group; 0.72 ± 0.20, oxidized fat group; 0.75 ± 0.31, clofibrate group; *P *= 0.201). Treatment of pregnant rats with oxidized fat resulted in 4.0-, 4.4-, 2.5-, 2.8- and 2.0-fold higher relative mRNA concentrations of ACO, CYP4A1, L-CPT I, MCAD, and LCAD, respectively, in the liver compared to control treatment (*P *< 0.05; Fig. 1). Pregnant rats treated with clofibrate had 4.8-, 8.3-, 2.0- and 1.9-fold higher relative mRNA concentrations of ACO, CYP4A1, MCAD, and LCAD, respectively, in the liver than control rats (*P *< 0.05), whereas the relative mRNA concentration of L-CPT I did not differ between both treatment groups. Fetuses of the oxidized fat group had 2.2-, 6.0-, 3.2- and 2.0-fold higher relative mRNA concentrations of ACO, CYP4A1, MCAD, and LCAD, respectively, in the liver than those of the control group (*P *< 0.05), whereas the relative mRNA concentration of L-CPT I did not differ between both treatment groups. Fetuses of the clofibrate group had 3.5-, 28-, 18- and 3.9-fold higher relative mRNA concentration of ACO, CYP4A1, L-CPT I, and LCAD, respectively, in the liver than those of the control group (*P *< 0.05), whereas the relative mRNA concentration of MCAD did not differ between both treatment groups.

### Relative mRNA concentrations of SREBP-1c and FAS in livers of pregnant rats and fetuses

**Figure 2:**
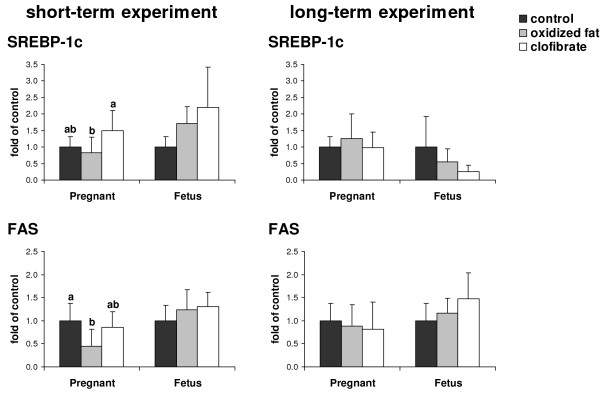
**Effects of treatment on relative mRNA concentrations of SREBP-1c and FAS in livers of pregnant rats and fetuses**. Effect of short-term (d 16 – d 21 of pregnancy, left) and long-term (d 1 – d 21 of pregnancy, right) administration of three different diets (control, oxidized fat, clofibrate) during pregnancy on relative mRNA concentrations of SREBP-1c and FAS in the liver of pregnant rats and fetuses at d 21 of pregnancy. Left, Bars represent mean ± SD (n = 9/group). Right, Bars represent mean ± SD (n = 12/group). Bars marked without a common superscript letter differ (*P *< 0.05). Results from one-way ANOVA (*P*-values): short-term experiment: SREBP-1c, *P *= 0.041 (pregnant), *P *= 0.058 (fetus); FAS, *P *= 0.026 (pregnant), *P *= 0.425 (fetus); long-term experiment: SREBP-1c, *P *= 0.526 (pregnant), *P *= 0.189 (fetus); FAS, *P *= 0.716 (pregnant), *P *= 0.334 (fetus).

In the short-term experiment, treatment of pregnant rats with oxidized fat resulted in lower relative mRNA concentrations of FAS in the liver relative to control treatment (*P *< 0.05), whereas the relative mRNA concentration of SREBP-1c did not differ between these two groups (Fig. 2). Relative mRNA concentrations of SREBP-1c and FAS in the liver of pregnant rats treated with clofibrate did not differ from those of control rats. However, pregnant rats treated with clofibrate had higher relative mRNA concentrations of SREBP-1c in the liver than those fed oxidized fat (*P *< 0.05). In fetal livers relative mRNA concentrations of SREBP-1c and FAS did not differ between treatment groups.

In the long-term experiment, relative mRNA concentrations of SREBP-1c and FAS in the liver of pregnant rats and fetuses did not differ between the three treatment groups (Fig. 2).

### Concentrations of triacylglycerols in livers of pregnant rats and fetuses

**Figure 3:**
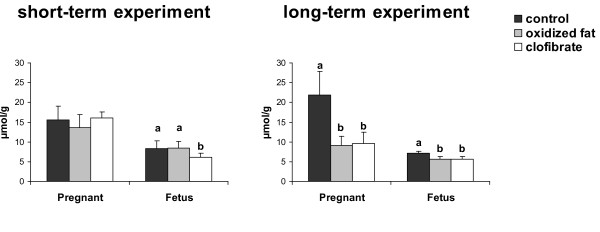
**Effects of treatment on triacylglycerol concentrations in livers of pregnant rats and fetuses**. Effect of short-term (d 16 – d 21 of pregnancy, left) and long-term (d 1 – d 21 of pregnancy, right) administration of three different diets (control, oxidized fat, clofibrate) during pregnancy on triacylglycerol concentrations in the liver of pregnant rats and fetuses at d 21 of pregnancy. Left, Bars represent mean ± SD (n = 9/group). Right, Bars represent mean ± SD (n = 12/group). Bars marked without a common superscript letter differ (*P *< 0.05). Results from one-way ANOVA (*P*-values): short-term experiment: *P *= 0.239 (pregnant), *P *= 0.025 (fetus); long-term experiment: *P *= 0.0001 (pregnant), *P *= 0.045 (fetus).

In the short-term experiment, concentrations of triacylglycerols in the liver of pregnant rats did not differ between the three treatment groups (Fig. 3). Fetuses of the clofibrate group had lower concentrations of triacylglycerols in the liver than those of the control and the oxidized fat group (*P *< 0.05); oxidized fat had no effect on the triaclyglycerol concentration in fetal livers compared to control.

In the long-term experiment, treatment of pregnant rats with oxidized fat or clofibrate resulted in lower concentrations of triacylglycerols in the liver compared to control treatment (*P *< 0.05). Fetuses of the oxidized fat group and the clofibrate group also had lower concentrations of triacylglycerols in the liver than those of the control group (*P *< 0.05).

### Relative mRNA concentrations of c-myc, c-jun, and c-fos in livers of pregnant rats and fetuses

**Figure 4:**
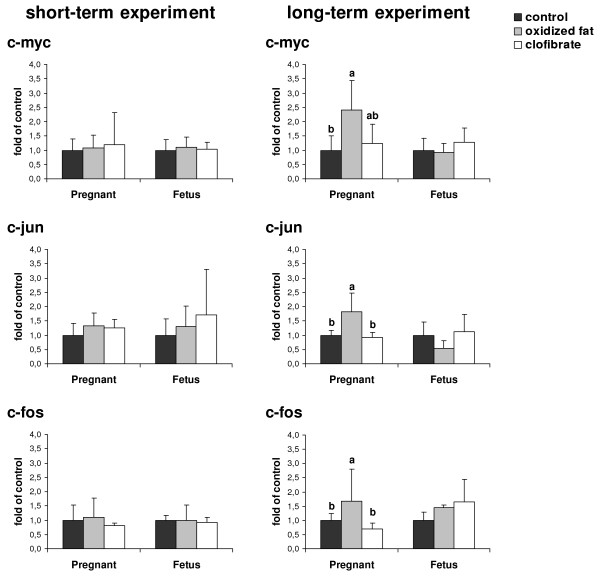
**Effects of treatment on relative mRNA concentrations of proto-oncogenes in livers of pregnant rats and fetuses**. Effect of short-term (d 16 – d 21 of pregnancy, left) and long-term (d 1 – d 21 of pregnancy, right) administration of three different diets (control, oxidized fat, clofibrate) during pregnancy on relative mRNA concentrations of c-myc, c-jun, and c-fos in the liver of pregnant rats and fetuses at d 21 of pregnancy. Left, Bars represent mean ± SD (n = 9/group). Right, Bars represent mean ± SD (n = 12/group). Bars marked without a common superscript letter differ (*P *< 0.05). Results from one-way ANOVA (*P*-values): short-term experiment: c-myc, *P *= 0.880 (pregnant), *P *= 0.807 (fetus); c-jun, *P *= 0.362 (pregnant), *P *= 0.552 (fetus); c-fos, *P *= 0.669 (pregnant), *P *= 0.966 (fetus); long-term experiment: c-myc, *P *= 0.001 (pregnant), *P *= 0.420 (fetus); c-jun, *P *= 0.0001 (pregnant), *P *= 0.086 (fetus); c-fos, *P *= 0.048 (pregnant), *P *= 0.323 (fetus).

In the short-term experiment, relative mRNA concentrations of c-myc, c-jun, and c-fos in the liver of pregnant rats and fetuses did not differ between the three treatment groups (Fig. 4).

In the long-term experiment, treatment of pregnant rats with oxidized fat resulted in higher relative mRNA concentrations of c-myc, c-jun, and c-fos relative to control treatment (*P *< 0.05). In fetal livers relative mRNA concentrations of c-myc, c-jun, and c-fos did not differ between treatment groups.

## Discussion

The present study demonstrates for the first time that components of oxidized fat with PPARα activating potential are able to induce a PPARα response in the liver of fetuses. Moreover, the present study shows that feeding oxidized fat during whole pregnancy, but not during late pregnancy, lowers triacylglycerol concentrations in fetal livers. Hydroxy- and hydroperoxy-fatty acids such as hydroxyoctadecadienoic acid (HODE) and hydroperoxyoctadecadienoic acid (HPODE) occurring in oxidized fats are very potent PPARα agonists [6,23,24]. These oxidized fatty acids are produced during the early stage of lipid peroxidation, and, due to their low thermodynamic stability, easily decompose at high temperatures. Thus, fats treated at low temperatures have markedly higher concentrations of these primary lipid peroxidation products than fats treated at high temperature [3]. Therefore, in order to provoke a significant PPARα activating effect of the oxidized fat, we decided to use a fat treated at a relatively low temperature for a long period. Although we did not determine the concentrations of oxidized fatty acids such as 13-HODE or 13-HPODE in the oxidized fat, the high peroxide value and the high concentration of conjugated dienes indicate that the oxidized fats used in both experiments presumably had high concentrations of hydroxy- and hydroperoxy-fatty acids which may be particularly responsible for the PPARα activating effect of oxidized fats. Nonetheless, other components of heated fats such as cyclic fatty acid monomers which also show a strong PPARα response [5] might be also causative for PPARα activation.

The lower concentrations of primary lipid peroxidation products (peroxides, conjugated dienes) in the oxidized fat used in the long-term experiment compared to that used in the short-term experiment are probably explained by the fact that in the long-term experiment the lipid peroxidation products in the oxidized fat were determined after inclusion into the diet. Primary lipid peroxidation products easily decompose and are partially degraded in the presence of other diet components such as metal ions (e.g. iron, copper) acting as catalysts.

Since unspecific effects might have been caused by a different fatty acid composition of the experimental fats (the heating process caused a loss of polyunsaturated fatty acids), we aimed at equalizing in particular the concentration of the polyunsaturated fatty acid C18:2 (n-6) in the fresh fat and the oxidized fat in the second experiment which lasted during the whole pregnancy. Although the concentrations of the long-chain saturated fatty acids C16:0 and C18:0 were consequently decreased in the oxidized fat compared to the fresh fat, we think that these differences are not responsible for the differences in the PPARα response observed between the experimental groups, because saturated long chain-fatty acids were shown to bind and activate PPARα only very weakly compared to polyunsaturated fatty acids such as C18:2 (n-6) [25]. Therefore, we assume that the PPARα response to an oxidized fat might depend on the balance between unoxidized fatty acids with low PPARα transactivation activity and oxidized fatty acids with high PPARα transactivation activity because both types of fatty acids compete for the PPARα-ligand binding site.

A further consequence of the use of lard in the control fat was that the cholesterol content of the control and the clofibrate diet slightly differed from that of the oxidized fat diet. However, based on an average cholesterol concentration of about 80 mg per 100 g lard the control and the clofibrate diet contained less than 0.004% cholesterol. Therefore, we assume that the slight difference in the cholesterol concentration between the experimental diets is negligible, especially since no relation between PPARα activation and dietary cholesterol is known from the literature.

The present study clearly shows that feeding oxidized fat during pregnancy, similar to clofibrate, which was used as a positive control, causes not only a PPARα response in the liver of pregnant animals as shown by liver enlargement and up-regulation of PPARα-responsive genes but also in the liver of the fetuses. Although the induction of the PPARα response by oxidized fat in the fetal liver was not as pronounced as observed with clofibrate, the oxidized fat also caused a strong up-regulation of PPARα-target genes of up to 9-fold in the livers of fetuses which was even more pronounced than the effect of oxidized fat in the livers of pregnant rats. Therefore, these findings suggest that not only pharmacological PPARα activators but also components of oxidized fat are able to sufficiently pass the placenta and activate PPARα in the fetal liver. This finding is novel since the placental transfer of these components of oxidized fat with PPARα activating potential from the maternal diet to the fetus is unknown. Indeed, the transplacental transport of fatty acids is highly selective for individual fatty acids [20-22], but no data are available from the literature with respect to the placental passage of oxidized fatty acids which are presumably decisive for the PPARα activating effect of oxidized fat. Thus, the present results indicate that these critical components of oxidized fats are also sufficiently transported across the placenta. Moreover, the observation from the short-term experiment that up-regulation of mRNA expression of ACO, CYP4A1 and L-CPT I by oxidized fat in the fetal liver was even more pronounced than in the liver of pregnant rats indicates that components of oxidized fat responsible for PPARα activation are presumably transported through the placenta with high preference.

Since activation of hepatic PPARα by clofibrate or oxidized fat has been demonstrated to enhance the fatty acid oxidation capacity in the liver and to lower hepatic triacylglycerol concentrations in non-pregnant rats [1,3,9], we also studied the effect of oxidized fat on the concentrations of triacylglycerols in the fetal liver. Indeed, we could demonstrate that feeding the oxidized fat during pregnancy also reduced the concentrations of triacylglycerols in the fetal liver indicating that the transplacental induction of PPARα responsive genes enhanced the peroxisomal and mitochondrial fatty acid oxidation capacity of the fetal liver. However, this effect has only been observed when the oxidized fat was fed during the whole pregnancy, but not when the oxidized fat was fed for the last 5 d of pregnancy only, although the PPARα responsive genes were markedly up-regulated in fetal livers of pregnant rats treated during either whole or late pregnancy. This suggests that short-term administration of oxidized fat to pregnant rats causes significant alterations on the gene expression level, which have no implications on the phenotypic level, e.g. triaclyglycerol concentrations. In contrast, short-term administration of clofibrate during pregnancy even revealed alterations on the phenotypic level as evidenced by reduced triacylglycerol concentrations in fetal livers. The latter might be attributed to the fact that clofibrate caused a more pronounced activation of PPARα in fetal livers due to a higher affinity for PPARα compared to oxidized fat leading to a marked induction of fatty acid oxidation and a significant lowering of triacylglycerol concentrations in fetal livers even after short-term exposure to clofibrate.

Interestingly, no effect of oxidized fat and even clofibrate on hepatic triacylglycerol concentrations following short-term treatment could be observed in pregnant rats, although clofibrate reduced triacylglycerol concentrations in fetal livers following short-term treatment. The failure of clofibrate or oxidized fat to reduce hepatic triacylglycerol concentrations in pregnant rats in the short-term experiment might be attributed to the fact that treatment was only performed during late pregnancy when significant alterations in lipid metabolism [e.g. hypertriacylglycerolemia as a consequence of enhanced adipose tissue lipolytic activity, enhanced liver production of VLDL particles, and decreased extrahepatic lipoprotein lipase activity [26-29]] occur in the pregnant animal. The enhanced arrival of free fatty acids, which also serve as ligands for PPARα, in the liver during late pregnancy could decrease the availability for the stronger PPARα activators clofibrate or components of oxidized fat with PPARα activating potential to their PPARα-ligand binding site. This in turn could reduce the capability of fibrates or oxidized fat to activate PPARα and consequently, its metabolic effects. This might be important since a substantial body of evidence suggests that not all alterations in gene transcription induced by pharmacological PPARα activators are also induced by fatty acids [30], e.g. some genes that contain a PPRE do not respond to fatty acids but to high-affinity PPARα activators [31-33]. Thus, administration of oxidized fat or clofibrate during late pregnancy might provoke differential effects on lipid metabolism than in virgin or early pregnant rats. The observation that treatment of rats with the triacylglycerol-lowering PPARα agonist fenofibrate during late pregnancy even increased triacylglycerol concentrations in plasma, whereas in virgin rats treatment with fenofibrate caused a reduction in plasma triacylglycerol concentrations [16], is probably supportive of this assumption.

Whether the effect observed with oxidized fat or clofibrate might have been also influenced by the metabolic state, e.g. fasting vs. non-fasting, cannot be answered with certainty. In the present study we decided to perform gene expression analysis of PPARα-responsive genes in the liver in the non-fasting state, because lipolysis of stored triacylglycerols in adipose tissue is strongly activated during fasting, resulting in a marked increase in plasma free fatty acid levels. These free fatty acids act as endogenous PPARα ligands and might have competed with the exogenous ligands (e.g. clofibrate, oxidized fatty acids or cyclic fatty acid monomers) for the PPARα-ligand binding site. Therefore, an altered ratio between endogenous free fatty acids and exogenous oxidized fatty acids as a consequence of the fasting state might have provoked a different PPARα response in the liver of pregnant rats and fetuses than observed in the non-fasting state. However, further studies are required to definitely resolve this question.

Only slight effects have been observed in the present study with respect to the lipogenic transcription factor SREBP-1c and its target gene FAS. Namely, oxidized fat caused a slight reduction in the mRNA abundance of FAS in the liver of pregnant rats, which is consistent with recent findings in non-pregnant rats [9]. However, no effect of oxidized fat has been observed on gene expression of lipogenic enzymes in fetal livers indicating that the reduced hepatic triacylglycerol concentrations in fetuses from pregnant rats treated with oxidized fat are probably largely due to an enhanced fatty acid oxidation capacity due to transplacental activation of PPARα. In part, the failure of oxidized fat on FAS gene expression in fetal livers might be explained by the fact that lipogenesis in fetal livers is generally very low and only increased during late pregnancy [34], and, therefore, probably does not respond to variations in the maternal diet.

Since pathological changes in newborn rats born to mothers treated with clofibrate during pregnancy have been reported [15], treatment with fenofibrate has been shown to impair fetal growth [16], and a link between induced CYP4A1 expression, peroxisome proliferation, and carcinogenesis in rat livers has been described [35], we also addressed possible adverse effects of treatment with oxidized fat on fetal livers. With respect to the hepatocarcinogenic effect of peroxisome proliferators in rodents it has been suggested that enhanced DNA synthesis as a consequence of up-regulation of proto-oncogenes including c-fos, c-jun, and c-myc might be mechanistically involved [17-19]. In the present study an up-regulation of proto-oncogenes in the livers of pregnant rats has been observed in the long-term experiment, but not in the short-term experiment, indicating that short-term administration of oxidized fat has no impact on mRNA expression of proto-oncogenes. However, in fetal livers mRNA expression of proto-oncogenes was not affected by oxidized fat regardless of the duration of oxidized fat administration suggesting that the oxidized fat is uncritical with respect to hepatocarcinogenesis. Unexpectedly, treatment with clofibrate had no effect on proto-oncogene expression in pregnant rats and fetuses either in the short-term and the long-term experiment, although it has been reported that the high-affinity PPARα-ligand WY-14,643 strongly up-regulated various proto-oncogenes in rat and mouse liver epithelial cells [18,19]. This differential action of WY-14,643 and clofibrate on proto-oncogene mRNA expression cannot be explained at the moment and, therefore, requires further research activities.

Although oxidized fat had no effect on proto-oncogene expression in fetal livers in the present study, oxidized fats might be considered critically in view of inducing oxidative stress in different tissues as shown in recent studies [36,37]. In addition, specific components of oxidized fats such as cyclic fatty acid monomers, which are formed in substantial amounts during domestic frying of frozen foods in sunflower oil [38], are probably toxic, e.g. earlier studies reported that mice receiving cyclic fatty acid monomers as well as rat pups from mothers fed cyclic fatty acid monomers had a higher death rate [39-41].

## Conclusion

In conclusion, the present study demonstrates for the first time that components of oxidized fat with PPARα activating potential contained in the maternal diet are able to induce a PPARα response in the liver of fetuses as evidenced by an up-regulation of PPARα target genes. In addition, the present study shows that feeding oxidized fat during whole pregnancy, but not during late pregnancy, lowers triacylglycerol concentrations in fetal livers, probably as a consequence of an enhanced peroxisomal and mitochondrial β-oxidation capacity. Although administration of oxidized fat during pregnancy had not impact on fetal proto-oncogene mRNA expression either after short-term or long-term administration, the observed pronounced transplacental PPARα activation by oxidized fat might be considered critically because of other recently reported adverse effects of treatment with PPARα activators during pregnancy [15,16]. Therefore, further research should be encouraged with respect to possible detrimental effects of oxidized fat on fetal development.

## Methods

### Animals

Two experiments were carried out with female Sprague-Dawley rats obtained from Charles River (Sulzfeld, Germany). At 11 wk of age, the rats were mated by housing one male rat with two female rats. D 1 of pregnancy was assigned upon observation of sperm in the vaginal smears, at which time rats were randomly assigned to the treatment groups. The short-term experiment was performed from d 16 to d 21 of pregnancy and included 27 pregnant rats with an initial body weight (d 16 of pregnancy) of 297 ± 26 (Mean ± SD) g, which were allotted to three groups of nine rats each. The long-term experiment was performed from d 1 to d 21 of pregnancy and included 36 rats with an initial body weight (d 1 of pregnancy) of 238 ± 27 g, which were allotted to three groups of twelve rats each. Pregnant rats were kept individually in Macrolon cages in a room maintained with controlled temperature (23 ± 1°C), humidity (50–60%), and lighting (0600 to 1800 h). All experimental procedures described followed established guidelines for the care and handling of laboratory animals [42] and were approved by the council of Saxony-Anhalt.

### Diets

#### Short-term experiment

In the short-term experiment, rats received 2 mL of different experimental fats by gavage daily at 0800 h, and, additionally, fed a commercial standard rodent diet (Altromin, Lage, Germany). To standardize food intake, the diets were fed daily in controlled amounts of 16 g per d. The first group (control group) received sunflower oil, the second group (oxidized fat group) oxidized fat (see "preparation of the oxidized fat"), and the third group (clofibrate group) sunflower oil containing 75 mg clofibrate (Fluka, Buchs, Switzerland) equivalent to 250 mg clofibrate per kg body weight. The experimental fats were given for 5 d from d 16 of pregnancy to d 21 of pregnancy. The standard diet was completely consumed by all the rats. Thus, all the rats within this experiment consumed identical amounts of the food.

#### Long-term experiment

In the long-term experiment, semipurified diets, composed according to the recommendations of ASNS for rats during reproduction [43], were used. The diet consisted of (g/kg diet): casein, 200; cornstarch, 390; saccharose, 198; cellulose, 50; fat, 100; mineral mixture, 40; vitamin mixture, 20; DL-methionine, 2. The type of fat was varied according to a one-factorial design. The first group (control group) received a mixture of sunflower oil and lard (54:46, w/w) was used. This ratio was chosen to equalize the fatty acid composition of the fresh fat with that of the oxidized fat, since the heating process caused a loss of polyunsaturated fatty acids, therefore, excluding that the treatment effects were caused by a different fatty acid composition of the experimental fats. The second group (oxidized fat group) received oxidized fat (see "preparation of the oxidized fat"). The third group (clofibrate group) received the same fat as in the control group, and clofibrate was added to the diet at a concentration of 5 g/kg. The vitamin E concentration of the diets was 50 mg α-tocopherol equivalents per kg diet. To adjust the vitamin E concentration of the diets, the native concentrations of tocopherols of the fats were analyzed. Based on the native concentrations of the fats, diets were supplemented individually with all-rac-α-tocopheryl acetate (the biopotency of all-rac-α-tocopheryl acetate is considered to be 67% of that of α-tocopherol). Diets were prepared by mixing the dry components with the fat and water and subsequent freeze drying. The residual water content of the diet was below 5 g/100 g of diet. In preliminary experiments rats fed diets with clofibrate or oxidized fat ad libitum consumed their diets over a longer period than rats fed control diets, which consequently shortened the fasting period and which itself has a pronounced effect on PPARα-response. Therefore, we decided to administer food daily at 0800 h in controlled amounts to standardize intake and to ensure that rats from all treatment groups had a comparable fasting period. The amount of food administered was 20% less than the amounts of identical diets with fresh fats consumed ad libitum by rats in preliminary studies. The amount of food offered daily was increased continuously during the experiment from 14 g to 17 g. In this feeding system, the food offered was completely consumed by all the rats. Thus, all the rats within this experiment consumed identical amounts of food. The experimental diets were fed from d 1 of pregnancy to d 21 of pregnancy.

In both experiments, water was available *ad libitum *from nipple drinkers during the whole experiment.

### Preparation of the oxidized fat

The oxidized fat was prepared by heating sunflower oil at a temperature of 60°C for 25 d. Sunflower oil was filled into a glass beaker and placed into a drying oven set at the intended temperature. Throughout the heating process, air was continuously bubbled through the fat at a flow rate of 650 ml/min. This treatment caused a loss of polyunsaturated fatty acids, and a complete loss of tocopherols and raised the concentrations of lipid peroxidation products in the fats. The extent of lipid peroxidation in the oxidized fat was estimated by assaying the peroxide value (POV) [44], acid value [44], concentration of thiobarbituric acid substances (TBARS) [45], and concentration of conjugated dienes [46]. To assess lipid peroxidation products in the oxidized fat after inclusion into the diet (long-term experiment), the fat was extracted from aliquots of the diets with a mixture of hexane and isopropanol (3:2, v/v) and analysed for peroxide value, concentration of conjugated dienes, and TBARS.

### Sample collection

4 h after the final portion had been administered the rats were anesthetized with diethyl ether and killed by decapitation. The liver and fetuses were excised immediately, and frozen with liquid nitrogen. In addition, livers from three randomly taken fetuses per pregnant rat were excised, and frozen with liquid nitrogen. All samples were stored at -80°C pending analysis.

### Lipid analysis

Lipids of maternal livers and pools of fetal livers were extracted with a mixture of hexane and isopropanol (3:2, v/v) [47]. Total cholesterol and triacylglycerol concentrations were determined using enzymatic reagent kits obtained from Merck Eurolab (Darmstadt, Germany). Prior to enzymatic measurement, lipids of the extract were dissolved in Triton X-100 as described by De Hoff et al. [48]. Fatty acid composition of experimental fats was determined by GC-FID analysis of fatty acid methyl esters (FAME) as described previously in detail [49].

### RNA isolation and real-time RT-PCR

For the determination of mRNA expression levels of PPARα, CYP4A1, ACO, L-CPT I, MCAD, LCAD, SREBP-1c, FAS, c-myc, c-jun, and c-fos total RNA were isolated from maternal liver and fetal liver pools using Trizol™ reagent (Invitrogen, Karlsruhe, Germany) according to the manufacturer's protocol. RNA concentration and purity were estimated from the optical density at 260 and 280 nm, respectively. cDNA synthesis and relative quantification of target gene mRNA compared to the housekeeping gene GAPDH mRNA was determined by real-time detection RT-PCR as described previously [50]. Sequences of gene-specific primers obtained from Operon (Köln, Germany) were as follows (NCBI GenBank; forward, reverse): GAPDH (NM_017008; 5'-GCA TGG CCT TCC GTG TTC C-3', 5'-GGG TGG TCC AGG GTT TCT TAC TC-3'), PPARα (NM_013196; 5'-CCC TCT CTC CAG CTT CCA GCC C-3', 5'-CCA CAA GCG TCT TCT CAG CCA TG-3'), CYP4A1 (M14972; 5'-CAG AAT GGA GAA TGG GGA CAG C-3', 5'-TGA GAA GGG CAG GAA TGA GTG G-3'), ACO (J02752; 5'-CTT TCT TGC TTG CCT TCC TTC TCC-3', 5'-GCC GTT TCA CCG CCT CGT A-3'), L-CPT I (NM_031559; 5'-GGA GAC AGA CAC CAT CCA ACA TA-3', 5'-AGG TGA TGG ACT TGT CAA ACC-3'), MCAD (NM_016986; 5'-CAA GAG AGC CTG GGA ACT TG-3', 5'-CCC CAA AGA ATT TGC TTC AA-3'), LCAD (NM_012819; 5'-AAG GAT TTA AGG GCA AGA AGC-3', 5'-GGA AGC GGA GGC GGA GTC-3'), SREBP-1c (XM_213329; 5'-GGA GCC ATG GAT TGC ACA TT-3', 5'-AGG AAG GCT TCC AGA GAG GA-3'), FAS (NM_017332; 5'-AGG TGC TAG AGG CCC TGC TA-3', 5'-GTG CAC AGA CAC CTT CCC AT-3'), c-myc (NM_012603; 5'-CTG GAG TGA GAA GGG CTT TG-3', 5'-CAG CAG CTC GAA TTT CTT CC-3'), c-jun (NM_021835; 5'-ACC AAG AAT TCC GTG ACG AC-3', 5'-CAA GGT CAT GCT CTG CTT CA-3'), and c-fos (NM_022197; 5'-CAT CGG CAG AAG GGG CAA AGT AGA G-3', 5'-TGC CGG AAA CAA GAA GTC ATC AAA G-3').

### Statistical analysis

Treatment effects were analyzed using one-way ANOVA. For significant *F*-values, means were compared by Fisher's multiple range test. Differences with *P *< 0.05 were considered significant.

## List of abbreviations used

ACO, acyl-CoA oxidase; CYP4A1, cytochrome P_450 _4A1; FAS, fatty acid synthase; LCAD, long-chain acyl-CoA dehydrogenase; L-CPT I, L-type carnitin-palmitoyl transferase I; MCAD, medium-chain acyl-CoA dehydrogenase; PPARα, peroxisome proliferator-activated receptor α; PPRE, PPAR response element; SREBP-1c, sterol regulatory-element binding protein-1c.

## Competing interests

The author(s) declare that they have no competing interests.

## Authors' contributions

RR participated in the design of the study and in the interpretation of the results and prepared the manuscript.

AG and CD carried out the feeding experiments, quantification of lipid concentrations, and mRNA expression analysis.

CB participated in the design and coordination of the study, and interpretation of the results.

KE conceived of the study and its design, coordinated work, participated in the interpretation of the results, and helped to draft the manuscript.

All authors read and approved the final manuscript.
